# The psychometric properties of the ADHD rating scale—5 for children and adolescents, home version for Sudan

**DOI:** 10.3389/fpsyt.2024.1365189

**Published:** 2024-06-11

**Authors:** Mohammed Al Jaffal, David Becker, Abdulkarim Alhossein, Salaheldin Farah Bakhiet, Rashed Aldabas, Abdulrahman Abaoud, Nagda Mohamed Abdu Elrahim, Hanan Alharthi

**Affiliations:** ^1^ King Saud University, College of Education, Department of Special Education, Riyadh, Saudi Arabia; ^2^ Chemnitz University of Technology, Department of Psychology, Chemnitz, Germany; ^3^ Sudan University of Science & Technology, College of Education, Department of Psychology, Khartoum, Sudan

**Keywords:** psychometric properties, Sudan, confirmatory factor analysis, home version, ADHD rating scale-5 for children and adolescents

## Abstract

There is a lack of universal scales for tracking ADHD symptoms in the home for children/adolescents in the Sudanese context. For this reason, this study aimed to validate the ADHD Rating Scale—5 for Children and Adolescents, Home Version for use by parents in Sudan to assess their children for ADHD. This scale is widely used by parents to assess their children aged 5–17 years for ADHD in the home environment. The current study involved 3,742 Sudanese parents of school-aged children and adolescents, each asked to complete the instrument for one child in their family; only one parent per family participated in the study. The authors then examined the psychometric properties of the scale from the completed assessments. The results indicated acceptable to high reliability for the total scale and both the symptom and impairment items. Exploratory and confirmatory factor analyses demonstrated high external and construct validity when applying the scale to the Sudanese sample. the factor structure resembled that of the normative U.S. sample in terms of the number of extractable factors and the strength of factor loadings. Based on the results, this adaptation of the home version of the ADHD Scale—5 for Children and Adolescents is both valid and reliable for use by Sudanese parents in the home environment.

## 1 Introduction

Attention-deficit/hyperactivity disorder (ADHD) is one of the most common neurodevelopmental disorders and comprises three major symptoms: inattention, hyperactivity, and impulsivity (1). Using the criteria outlined in the *Diagnostic and Statistical Manual of Mental Disorders* (5th ed.; DSM-5; 2), studies have estimated that the global rates of ADHD among children are from 2% to 7% (with a ~5% average) and determined that the condition affects from 2.58% to 6.76% of adults worldwide (3, 4). Similarity, a recent meta-analysis study revealed that approximately 7.6% of children aged 3 to 12 years and 5.6% of teenagers aged 12 to 18 years have ADHD. These prevalence rates are higher than those reported in previous studies using different diagnostic criteria (5). As a result, there has been a rapid increase in the number of patients receiving treatment for ADHD across all age group (6). According to the Centers for Disease Control and Prevention (7) data collected between 2016–2019 indicate that male children in the United States are more than twice as likely to receive an ADHD diagnosis compared to their female peers.

The exact cause of ADHD is unknown, but it is believed that neurological factors that affect the brain’s structure may be behind the condition (8). Symptoms of ADHD significantly affect an individual’s social, academic, and professional function, particularly when early diagnosis is lacking (9). Consequently, researchers emphasize the importance of early diagnosis to support both children and their parents in terms of seeking necessary and appropriate services (10, 11). It is recommended that diagnosis includes monitoring for established symptoms of ADHD over a 6-month time period and that this monitoring occurs at two or more sites (e.g., school and home; 12). Parental involvement is a crucial aspect of early intervention for the condition (1).

In recent years, Sudan has witnessed an increase in the prevalence of ADHD among children and adolescents (13). Despite research in other countries underscoring the pivotal role of parents in mitigating the impact of the condition, there is a scarcity of studies on parent-rated measures of ADHD in Sudan. One notable effort identified during our literature review is the study by El-Hassan Al-Awad and Sonuga-Barke (14), an early investigation that examined both parent- and teacher-driven models for assessing ADHD. This study utilized Conners’ Rating Scales, introduced in the early 1970s, which are based on checklists allowing teachers to assess the efficacy of drug therapy for behavioral disorders (15, 16). Although this series of scales remains in use for ADHD assessment, the version employed in the El-Hassan Al-Awad and Sonuga-Barke study is now 25 years old and differs from the one examined in current research. While the scales demonstrated high levels of reliability and internal consistency in their study involving a stratified sample of 300 families with children aged 6–10 years, the dynamic nature of ADHD rates in Sudan necessitates updated investigations. Subsequent research in Sudan has predominantly focused on validating teacher-rated measures of ADHD (e.g., Madani A (2007)^1^, Fathi J (2010)^2^, Khalaf Allah K (2011)^3^ 17). Certain pediatric researchers in Sudan have explored various aspects of ADHD diagnosis, examining its impact on parents and the influence parents can exert on children with ADHD (e.g. Mohammed H (2014)^4^, 9, 18). These studies have utilized international ADHD scales. For instance, Mohammed H (2014)^4^ translated and validated the Weiss Functional Impairment Rating Scale (WFIRS-P) for use in Sudan, administering it to 120 parent-child pairs visiting clinics to assess socio-demographic characteristics and functional impairment levels. Osman et al. (9) validated the Swanson, Nolan and Pelham Teacher and Parent Rating Scale (SNAP-IV) for assessing ADHD and oppositional defiance disorder (ODD) in adolescents/young adults and children. Although their research included a pilot study with 50 students, detailed information on the validity and reliability of their results was not provided.

A plurality of measurement instruments can assist in testing convergent validity. Hence, the availability of another valid ADHD scale for Sudan would be desirable. The ADHD Rating Scale—5 for Children and Adolescents has already been translated into Arabic and applied in Saudi Arabia by Alhossein and Bakhiet (19). This version is intended to be used here as well, however, the linguistic similarities between Sudan and Saudi Arabia do not diminish the necessity for a revalidation due to significant cultural, historical and ethnic differences.

Due to the aforementioned drawbacks to the existing literature on measures of ADHD for the Sudanese context, this study aimed to bridge the identified gaps by investigating the psychometric properties of the ADHD Rating Scale—5 for Children and Adolescents, Home Version (ADHD Rating Scale—5; 20) using a large sample of children and adolescents from Sudan. The aim of this study is to examine the reliability and diagnostic validity of the ADHD Rating Scale—5 for this country. To achieve this, the knowledge gained from the Western normative sample regarding reliability and factor structure will be attempted to be replicated, as well as the results obtained from the school version for Sudan (13). It is assumed that there will be a high congruence between the findings of this study and those of previous studies. Such a congruency is of fundamental importance because, despite compulsory education and state-funded schooling, Sudan still suffers from an extremely low enrollment rate, especially in rural areas. The ability to diagnose based on data collected by parents themselves is therefore of greater significance in Sudan than in Western countries. Furthermore, a general improvement in diagnostic validity is advisable, as mis- and overdiagnoses of ADHD can have significant consequences for the individuals affected as well as their families (21–23).

## 2 Methods and procedures

This study employed a descriptive research design with some confirmatory elements to assess the psychometric properties of the ADHD Rating Scale—5 for use in Sudan (24). Approval to conduct the research was obtained from Ministry of Education in Sudan. To ensure effective implementation and understanding of the scale among parent participants, thirty-five graduate students from the University of Khartoum were recruited to provide support throughout the research process. A workshop was then presented to these 35 individuals regarding completing, correcting, and grading the scale. This workshop also taught the grad students how to present the scale to the parents, including instructions on how to explain the process of completing the scale. Next, to facilitate the recruitment process, the 35 graduate students were divided into seven groups of five and directed to contact schools located in the seven localities of each of Khartoum.

### 2.1 Subjects and participants

The stratified random sampling method was employed to ensure that the study participants represented the full spectrum of demographic characteristics of families with children in schools across three cities in Sudan. Graduate students then provided potential participants with information regarding the study’s purpose, participation process, and a copy of the Consent Form. Alongside the form, participants received a copy of the scale to fill out upon consenting to participate. Prior to enrollment, all potential participants were informed that participation was entirely voluntary, and they retained the right to withdraw from the study at any time. The study involved 3,742 school-aged Sudanese children aged from 5–17 where one parent of each completed the home version of the scale. Of these individuals, 1,947 (52.03%) were male and 1,795 (47.97%) were female. There were 1,288 (34.42%) children who lived in the capital city of Khartoum, 1,012 (27.04%) who resided in Bahri, and 1,442 (38.54%) who were from Omdurman. There were 1,392 (37.20%) subjects who were 13–17, 1,247 (33.32%) who were 9–12, and 1,103 (29.48%) who were aged 5–8 years. The majority (68.81%) of participants were enrolled in elementary school (*n* = 2,575), 862 (23.04%) were in secondary school, and 305 (8.15%) were in preschool. Only 56 (1.50%) of the 3,742 participants had been diagnosed with ADHD.

For the purposes of the study, we asked that only one parent complete the scale for each child subject, therefore the total number of parent respondents was also 3,742. Of these parents, 1,765 (47.17%) were male and 1,977 (52.83%) were female; they were aged from 20–87 years (*M* = 42.11; *SD* = 8.78). The educational achievement of the parents was identified as: (a) 1,896 (50.67%) had a high school diploma or less; (b) 1,509 (40.33%) had a bachelor’s degree; and (c) 337 (9.00%) had a master’s degree. Parental annual income, indicated in Sudanese pounds, were categorized as: (a) low-income (less than £20,000): 1,467 (39.20%); (b) mid-income (£20,000–£50,000): 1,704 (45.54); (c) high-income (greater than £50,000): 571 (15.26%). There were 438 (11.70%) parents who attended a special program: (a) 153 (4.09%) attended one of these, (b) 119 (3.18%) attended two of these, (c) 46 (1.23%) attended three of these, and (d) 120 (3.21%) attended more than three special programs.

### 2.2 Instrument

The ADHD Rating Scale-5 (20) is a comprehensive assessment tool used to evaluate symptoms and impairment in children according to the DSM-5 criteria. This scale consists of eighteen items and focuses on two primary domains: inattention (abbreviated as “Inatt.”) and hyperactivity-impulsivity (abbreviated as “Hyp.-Imp.”). The inattention subscale comprises the following items: Attention to details, sustaining attention, appearing not to listen, following instructions, difficulty organizing, sustained mental effort, losing things, being easily distracted, and forgetfulness. The hyperactivity-impulsivity subscale includes the following items: Fidgeting, leaving seat, running around, playing quietly, being constantly on the go, excessive talking, blurting out answers, waiting for turns, and interrupting or intruding. Respondents are asked to rate each item on a 4-point Likert scale, ranging from 0 (“never or rarely”) to 3 (“very often”). To determine the total scores for the respective (sub)scales, the scores of all associated items are summed.

The process of translating the scale went through several stages. Initially, the research team translated all items of the scale. Subsequently, these items were juxtaposed with their English counterparts. They were then reviewed by a group of specialists with doctorate degrees in special education, who also served as university faculty members and were proficient in English, to assess the validity and accuracy of the translation. Following their review, several comments were provided on the initial translation. These comments were then utilized to revise and improve certain items to achieve an appropriate version of the scale. Once the revisions were completed, the scale was presented in its final form to another group of specialists to evaluate its clarity and suitability for the Sudanese environment. The respondents indicated that the scale’s paragraphs were suitable for use in the Sudanese context.

### 2.3 Data collection and analysis procedures

The data collection process spanned approximately one month. Throughout this period, a team of 35 graduate students was organized into seven groups, with each group consisting of five students. Their primary objective was to establish contact with various schools in Khartoum in order to recruit potential participants. All analyses described within this section were done with the free software R (Version 4.0.0; 25), using the packages and settings as follows. To meet the objectives of the study, we first ran reliability analyses, using Cronbach’s α, Guttman’s 6, and McDonald’s ω from R-package Psych (Version 2.2.5; 26, 27; Zinbarg). In the second step, we ran exploratory factor analyses (EFA) using the R-packages Lavaan (Version 0.6–7; 28), ltm (Version 1.1–1; 29) nFactors (Version 2.4.1; 30), and psy (Version 4.0.3; 31). Before this, we checked our data for common variance with overall and single-item measure of sampling adequacy (MSA) by Kaiser-Meyer-Olkin factor adequacy (KMO). In EFA, we used oblimin for rotation as more than one factor was expected, and, under the assumption of factor intercorrelations, we used maximum likelihood (ML) as the estimator. Decisions about extracted number of factors were done using the parallel analysis approach, the optimal coordinates approach, and Velicer MAP. However, we also ran confirmatory factor analyses (CFA) to compare factor structures from the data to those from DuPaul, Power, et al. (20).

## 3 Results

### 3.1 Reliability and sampling adequacy

DuPaul, Power, et al. (20) assumed more than one factor for the nine symptom items, thus reliability was estimated for the full ADHD Rating Scale—5 as well as the two subscales Inattention and Hyperactivity-Impulsivity, separately. Single item drops did not lead to changes in reliability beyond the second decimal place. Also, results from the different measurements of reliability are mostly high (>.80) or at least acceptable for both scales of the symptom and also of the impairment items (see **Tables 1** and **2**). Furthermore, the KMO Test consistently certifies MSAs >.90 for the overall symptom-scales (.95) as well as single symptom-items (.93 to.97), and at least >.80 for the impairment-scales (.88) and impairment-items (.85 to.90). Thus, no item had to be excluded for factor analyses.

**Table 1:** Reliability for the Symptom Items and Item Drop of the ADHD Rating Scale-5, Home Version.

Item	*N*	Cronbach’s α						McDonald’s ω	
		raw	Std	95% CI		G6	*M_r_*	Hierarch.	Total
				*LL*	*UL*				
Drop: Attention to details	3742	.85	.84	–	–	.84	.42	–	–
Drop: Sustaining attention	3742	.85	.84	–	–	.84	.42	–	–
Drop: Does not seem to listen	3742	.86	.84	–	–	.84	.43	–	–
Drop: Follow instructions	3741	.85	.83	–	–	.83	.41	–	–
Drop: Difficulty organizing	3742	.85	.83	–	–	.83	.41	–	–
Drop: Sustained mental effort	3742	.85	.84	–	–	.84	.42	–	–
Drop: Loses things	3742	.86	.85	–	–	.85	.43	–	–
Drop: Distracted	3742	.85	.83	–	–	.83	.41	–	–
Drop: Forgetful	3742	.85	.84	–	–	.84	.42	–	–
Inatt. Total	3742	.87	.87	.86	.87	.86	.42	.83	.89
Drop: Fidgets	3742	.85	.85	–	–	.84	.40	–	–
Drop: Leaves seat	3742	.85	.85	–	–	.84	.39	–	–
Drop: Runs about	3742	.84	.84	–	–	.83	.38	–	–
Drop: Playing quietly	3742	.85	.85	–	–	.84	.40	–	–
Drop: On the go	3742	.84	.84	–	–	.83	.37	–	–
Drop: Talks excessively	3741	.84	.84	–	–	.83	.39	–	–
Drop: Blurts out answers	3740	.84	.84	–	–	.83	.39	–	–
Drop: Awaiting turns	3742	.84	.84	–	–	.83	.38	–	–
Drop: Interrupts or intrudes	3742	.85	.85	–	–	.84	.39	–	–
Hyp.-Imp. Total	3742	.86	.86	.85	.87	.85	.39	.77	.88

Analyses done for Inatt. and Hyp.-Imp. separately; A = Inatt., H = Hyp.-Imp.; N varies due to missing/false values; reliability for Inatt. and Hyp.-Imp., lower and upper α for 95% confidence boundaries, G6 = Guttman’s 6, M_r_ = mean intercorrelation; results for items: if dropped.

**Table 2:** Reliability for the Impairment Items and Item Drop of the ADHD Rating Scale-5, Home Version.

Item	*N*	Cronbach’s α						McDonald’s ω	
		raw	Std	*LL*	*UL*	G6	*M_r_*	Hierarch.	Total
Drop: Teacher relations	3742	.82	.82	–	–	.79	.47	–	–
Drop: Peer relations	3741	.82	.82	–	–	.79	.48	–	–
Drop: Academic functioning	3742	.81	.81	–	–	.78	.47	–	–
Drop: Behavioral funct.	3742	.81	.81	–	–	.78	.46	–	–
Drop: Homework funct.	3742	.81	.81	–	–	.78	.47	–	–
Drop: Self-Esteem	3742	.80	.81	–	–	.78	.45	–	–
After Inatt. Rating	3742	.84	.84	.83	.85	.82	.46	.76	.87
Drop: Teacher relations	3742	.84	.84	–	–	.81	.52	–	–
Drop: Peer relations	3742	.85	.85	–	–	.82	.52	–	–
Drop: Academic funct.	3742	.83	.84	–	–	.81	.51	–	–
Drop: Behavioral funct.	3742	.84	.84	–	–	.81	.51	–	–
Drop: Homework funct.	3742	.84	.84	–	–	.82	.51	–	–
Drop: Self-Esteem	3742	.83	.84	–	–	.82	.49	–	–
After Hyp.-Imp. rating	3742	.86	.86	.85	.87	.85	.52	.77	.91

Analyses done for Inatt. and Hyp.-Imp. separately; A = Inatt., H = Hyp.-Imp.; N varies due to missing/false values; reliability for Inatt. and Hyp.-Imp., lower and upper α for 95% confidence boundaries, G6 = Guttman’s 6, M_r_ = mean intercorrelation; results for items: if dropped.

### 3.2 Symptom subscale factor analyses

**Figure 1:**
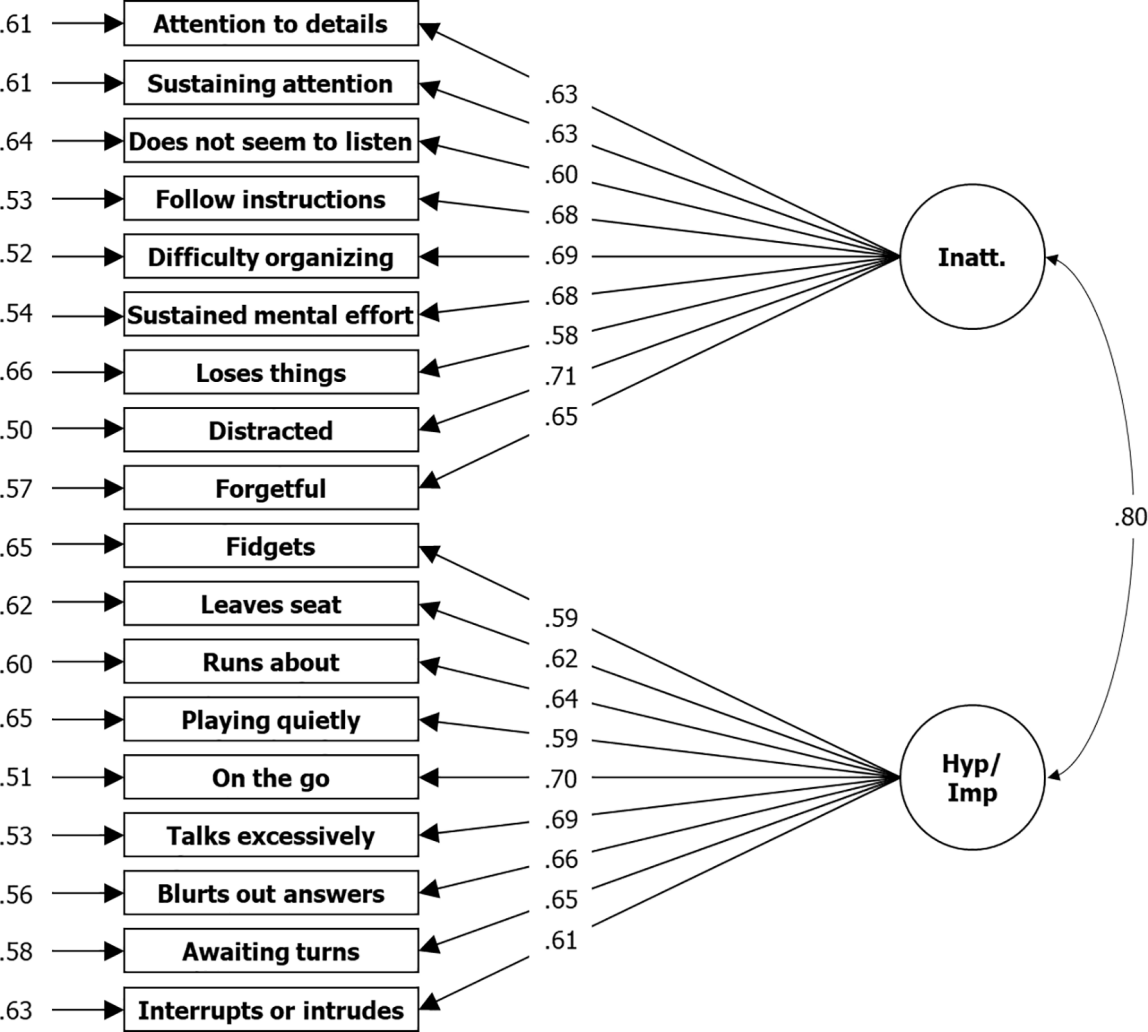
Results From CFA for Two Factor Solution Based on Symptom Items of the ADHD Rating Scale—5, Home Version. *N* = 3,739; Loglikelihood user model (H0) = –63766.953; Fits: (χ²[134] = 1323.670, CFI = .951, TLI = 0.944, RMSEA = .049, SRMR = .033; left: residual variances, center: factor loadings (lambda), right = correlation.

**Figure 2:**
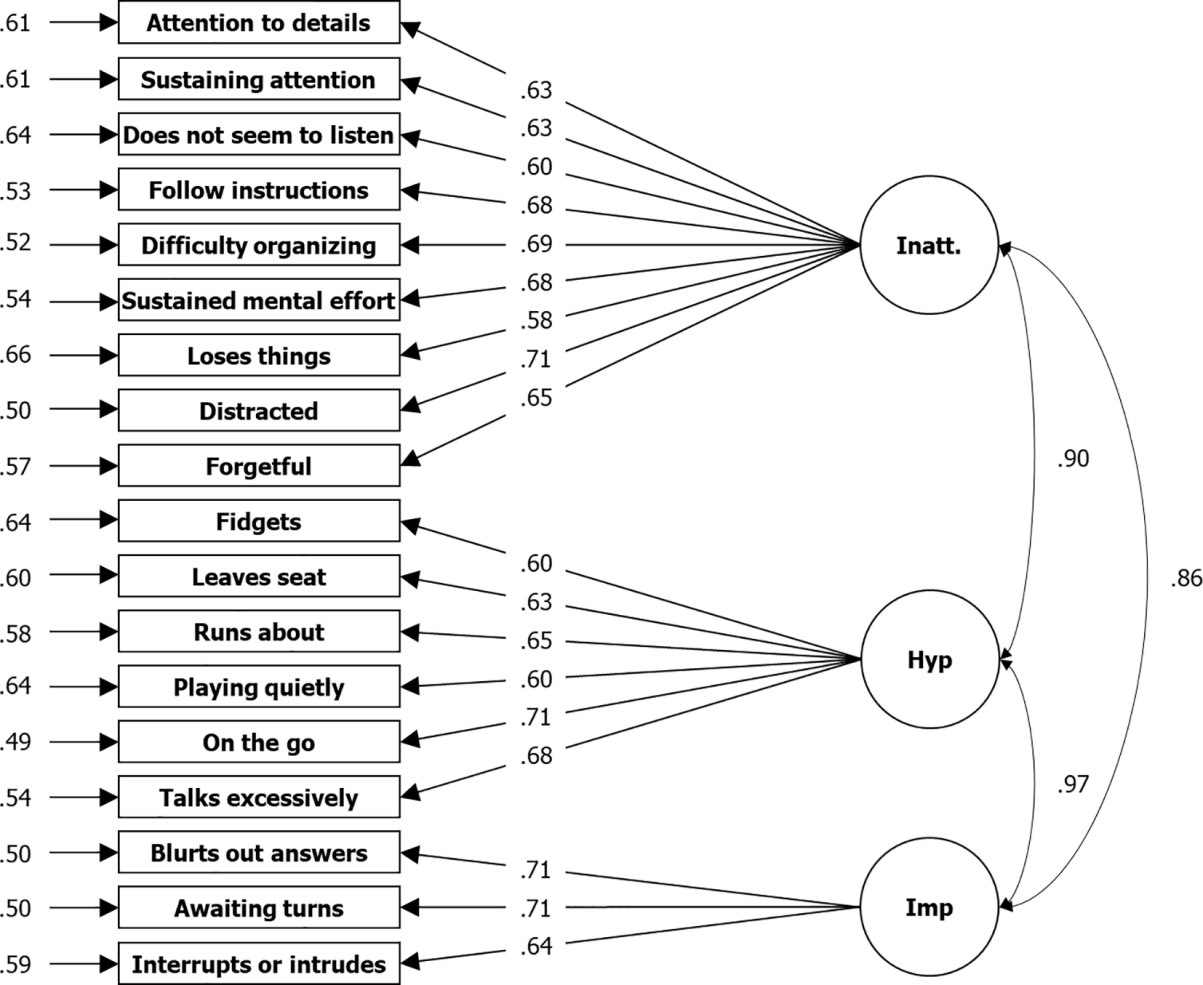
*Results From CFA for Three Factor Solution Based on Symptom Items of the ADHD Rating Scale—, Home Version*. *N* = 3,739; Loglikelihood user model (H0) = –63673.157; Fits: (χ²[132] = 1136.078, CFI = .959, TLI = 0.952, RMSEA = .045, SRMR = .031; left: residual variances, center: factor loadings (lambda), right = correlation.

For the symptom subscales, all three approaches used to determine the number of factors from EFA refer to a bifactorial solution, which obtained a good RMSR of.03 and *R*² of.52 for the first factor and.48 for the second factor, which cumulatively means 100% of variance explained. In congruency, two correlating factors are assumed by CFA without the assumption of an additional first order factor. Compared to a single factor solution, RMSEA is.05 instead of.09 and CFI is.96 instead.85. However, a three-factor solution also gained an RMSEA of.05 and CFI of.96 (see **Table 3**, **Figures 1**, **2**). It is clearly shown that factor structure within the data is congruent to the theoretical structure. Items measuring Inattention loading on one and items measuring Hyperactivity-Impulsivity on another factor in the two-factors solution, or items measuring Inattention loading on one and items measuring Hyperactivity-Impulsivity on two factors each Hyperactivity and Impulsivity in the three factors solution, congruent to the theory. However, in contrast to the bifactorial model from DuPaul, Power, et al. (20), we found lower factor loadings (λ = .59 to.71 compared to.75 to.91) across all items but a similar correlation (*r* = .75 compared to.80) between the two factors extracted. In the model with three factors, the correlation between factors for hyperactivity and impulsivity is nearly perfect (*r* = .97), which makes the need for such a subdivision questionable.

**Table 3:** EFA-Results for the Symptom Items of the ADHD Rating Scale-5, Home Version.

Items	Cmnl.	Factor loadings (λ)	
		C1	C2
Attention to details	.40	.66	–.04
Sustaining attention	.40	.61	.04
Does not seem to listen	.36	.54	.09
Follow instructions	.48	.74	–.07
Difficulty organizing	.49	.72	–.03
Sustained mental effort	.46	.68	.00
Loses things	.33	.47	.15
Distracted	.49	.60	.13
Forgetful	.42	.62	.04
Fidgets	.34	.15	.47
Leaves seat	.37	.15	.50
Runs about	.39	.10	.56
Playing quietly	.34	.17	.46
On the go	.50	–.02	.72
Talks excessively	.52	–.09	.78
Blurts out answers	.45	.02	.66
Awaiting turns	.43	–.02	.67
Interrupts or intrudes	.37	.06	.57
*R*²		.52	.48

Cmnl. = communalities; rotation = oblimin, extraction = Maximum Likelihood; N = 3,739, RMSR = .03.

### 3.3 Impairment subscale factor analyses

**Figure 3:**
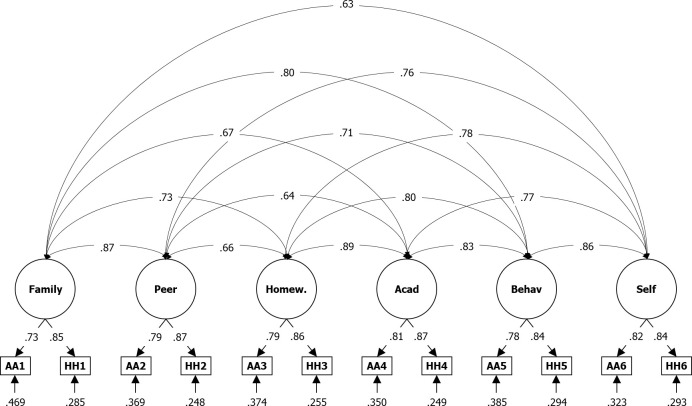
*Results From CFA for Six Factor Solution Based on Impairment Items of the ADHD Rating Scale—, Home Version*. IA = impairment item filled after Inatt. assessment, IH = impairment item filled after Hyp.-Imp. assessment; *N* = 3,741; Loglikelihood user model (H0) = –31573.074; Fits: (χ²[39] = 1641.468, CFI = .938, TLI = 0.896, RMSEA = .105, SRMR = .030; bottom: residual variances, center: factor loadings (lambda), top = correlation.

Similar to the symptom subscale, the optimal coordinates and the parallel analysis approaches both recommended a bifactorial solution for the impairment subscale, which results in an unsatisfying RMSR of.06. Indeed, Velicer MAP recommended a single-factor solution, but this deteriorated the RMSR to.09. Assuming a model with six factors instead, as shown in the manual of the ADHD-rating scale (20), we gained a highly satisfying RMSR of.02, in which all six factors together explain 100% of the variance. Thus, only the six-factor solution was used in the CFA (see **Table 4** and **Figure 3**). However, in this case, we did not gain satisfying fits, with a RMSEA of.09 and a CFI of.85, and factor loadings were much lower compared to those in the manual (λ = .59 to.71 compared to.73 to.87), even if the intercorrelations between the six factors extracted are similar (*r* = .63 to.89 compared to.60 to.94).

**Table 4:** EFA-Results for the Impairment Items of the ADHD Rating Scale-5, Home Version.

Items	Cmnl.	Factor loadings (λ)					
		C1	C2	C3	C4	C5	C6
Attention to details	.42	.01	.00	.00	.02	.02	.89
Sustaining attention	.69	.02	.08	.56	–.04	.00	.25
Does not seem to listen	.53	.01	.24	.00	.50	–.02	.17
Follow instructions	.64	.00	.99	.02	.00	.01	.00
Difficulty organizing	.46	.48	.20	–.01	.00	.04	.19
Sustained mental effort	.62	.06	.14	.03	.01	.49	.20
Fidgets	.52	.13	–.08	.28	.12	.11	.39
Leaves seat	.68	.00	.00	1.00	.01	.01	–.04
Runs about	.57	.01	–.02	.01	1.00	.01	–.01
Playing quietly	.66	.16	.45	.04	.25	.11	–.04
On the go	.52	1.01	–.02	.01	.00	.00	–.02
Talks excessively	.73	–.01	–.01	.00	.00	1.00	–.02
*R*²		.17	.17	.17	.17	.16	.15

Cmnl. = communalities; rotation = oblimin, extraction = Maximum Likelihood; N = 3,741, R²_cumul._ = 1.00, RMSR = .05.

## 4 Discussion

Reliability and factor structure of the ADHD-Rating Scale were conducted on the same sample as in the present study by Alhossein, et al (13) but using the school version, thus teachers instead of parents as raters. Within this section, the researchers want to focus on a comparison between their results and those from Alhossein, et al. (13) in the context of the results given by DuPaul, Power, et al. (20) and DuPaul, Reid, et al. (32) showing that the use of one or the other versions results in similar reliability and factor structure.

Initially, what we found for the symptom items was a noticeably lower reliability in the home compared to that of the school version (Inatt. total|Hyp.-Imp. total: α = .87|.86 vs.92|.90), where the differences also exceeded the respective 95% confidence intervals of around ± .01. This is true for the total scales and consistently across the changes in α for all items if dropped. However, the same pattern was found by DuPaul, Reid, et al. (32) in their comparison (Inatt. total|Hyp.-Imp. total: α = .93|.91 vs.96|.95). The same was found for the factor loadings, which are higher for the school version both in the Sudanese sample (bi-factorial model: λ = .65 to.80 vs.58 to.71) and in the U.S. normative sample (bi-factorial model: λ = .86 to.94 vs.75 to.91; (20), **Figure 2**). Differences also can be found regarding the bi-factorial model fits. Whereas DuPaul, Power, et al. (20) reported better CFI and TLI but worse RMSEA from the application of the scale in schools (CFI = .994 vs.993; TLI = .993 vs.991; RMSEA = .057 vs.040), all three fit-indices were worse for the Sudanese school application (CFI = .946 vs.951; TLI = .939 vs.944; RMSEA = .063 vs.049).

DuPaul, Power, et al. (20) give no reliabilities for impairment scores whereas in the Sudanese sample, Cronbach’s alpha from the school and home versions just slightly deviated and stayed within the 95% confidence intervals (Inatt. total|Hyp.-Imp. total: α = .87|.87 vs.84|.86). Factor loadings in the six-factor model from the U.S. sample show virtually no differences between teachers and parents (λ = .93 to.99 vs.92 to.98). This is also the case for the Sudanese sample (λ = .74 to.83 vs.73 to.87). Fit indices are slightly worse for application at schools compared to application at home both in the United States (CFI = .995 vs.997; RMSEA = .076 vs.050) and the Sudanese sample (CFI = .932 vs.938; RMSEA = .112 vs.105). In summary, although the gained values for reliability, factor loadings, and fits are lower or worse with the Sudanese sample as compared to the U.S. sample, the differences between teachers and parents follow the same pattern in both applications.

It is well-established in the literature that family participation in identifying and diagnosing children with disabilities is crucial. Parents can provide specialists with valuable data about their children’s behavior. Diagnosis cannot yield reliable results without the inclusion of parents’ insights and evaluations of their children’s behavior (33, 34). Given that parents are the most aware of their children’s behavior, the significance of parental assessment when collecting data on children and adolescents with ADHD should not be underestimated (e.g., 32, 35).

It is crucial to characterize and quantify children’s behaviors and symptoms in the home environment as this setting can provide insights not apparent in the school environment (36, 37). Despite the recognized importance of parental scales, there were previously a lack of standardized scales suitable for the Sudanese context to identify children with ADHD. This provided the impetus for our research, which aimed to extract indices of validity and reliability from the ADHD Scale-5 for Children and Adolescents, Home Version that is based on the *DSM-5* diagnostic criteria for ADHD (2).

Our adaptation of the scale demonstrated reliability in the Sudanese context. Correlations among items and their respective dimensions and the total score were calculated to establish the scale’s internal consistency. All correlations were significant (*p* ≤.05 and.01). This finding aligns with DuPaul’s (38) report of good internal consistency for the ADHD Rating Scale-5. We employed confirmatory factor analysis to establish the construct validity of the Sudanese version of the ADHD Rating Scale-5. This analysis revealed the scale to have a two-factor structure, a finding that is consistent with previous investigations of not just the ADHD Rating Scale-5 but other ADHD scales. This finding is also consistent with DuPaul et al. (39), (2016), which established the conceptual validity of the ADHD Rating Scale-5. We also established the scale’s reliability through Cronbach’s α and McDonald’s coefficients; the reliability coefficients for the two dimensions were 0.85–0.87 and 0.77–0.89, respectively. All reliability coefficients were high, a finding that agrees with those of previous studies (e.g., 39, 2016).

While our study demonstrated that both reliability and construct validity are also satisfied when utilizing the ADHD Rating Scale-5 for Children and Adolescents with parent ratings, our results raised some concerns about the usefulness of the involvement of parents in processes of psychological screening and diagnosis, as both criteria were overall better fulfilled by the teachers’ ratings (13). In particular this applies as parent involvement can cause negative side effects as concerns, uncertainty until anxiety, if they are not familiar with the issue (40–42). However, as the same studies show somewhat positive effects of family involvement in screening and diagnosis were educated about their children’s problems, their findings do imply the exclusion of the parents not necessarily, but an involvement after prior information and permanent support by a trained psychological staff. Moreover, considering the limited knowledge about the positive impact of parents in care and treatment of these children is little secured (43). Therefore, a decision regarding this issue should be based on a cost-benefit analysis that includes parental characteristics.

### 4.1 Study delimitations

Our study is limited by a number of factors. First of all, we only included parents of children and adolescents from 5 to 17 years of age in Khartoum, which means that our findings might not be generalizable to children in that age range who live in other areas of the country. Therefore, there is a need also to administer the scale in other cities and villages to reach a larger and more representative sample of Sudanese children and adolescents. Our findings should also be compared to those in the United States and Saudi Arabia, using the relevant versions of the scale for each, to identify the differences between them and the influence of cultural and social factors on symptoms. In addition, our findings cannot necessarily be applied to individuals with ADHD who are either older or younger than our established age range. In addition, we employed confirmatory factor analysis in our study to establish the construct validity of the scale; other types of validity need to be established in future investigations, e.g., discriminatory validity. Another point to criticize is the exclusive use of a single scale to assess ADHD, leading to a lack of examination regarding convergent validity. The next step should be to do this, as now there are two suitable instruments available: the SNAP-IV-C from the study by Osman et al. (9) and the ADHD Rating Scale-5 from this study.

### 4.2 Implications

A number of implications can be drawn from the results of our study. First, the ADHD Rating Scale-5 can be reliably used to identify ADHD in Sudanese children and adolescents. Second, it is important to include parents in the process of identifying ADHD in children, from which it can be inferred that parents should be trained on how to use the ADHD Rating Scale-5 to identify symptoms of ADHD in their children from age 5 to 17 years. It is also recommended that parents be encouraged to cooperate with schools to expand upon their children’s diagnoses and secure the appropriate interventions for them. Fourth, the ADHD Rating Scale—5 can be employed to assess the impact of interventions provided to children. Third, the ADHD Rating Scale-5 should be employed to conduct a survey to identify the prevalence of children and adolescents with ADHD in Sudan. Indeed, regarding the as yet untested convergent validity, it is advisable to use a second instrument in future studies, either on the same individuals or by split sampling method. Finally, the scale is inexpensive and easy to apply to obtain valuable data on ADHD symptoms in children and adolescents in the home environment. Our adapted scale for Sudan can be used by parents to regularly assess their children and thereby be able to recognize any changes that may appear in their behavior outside of school. Finally, the scale can be used to identify the effectiveness of interventions provided to children at school and home.

### 4.3 Conclusion

The study estimated the reliability of the ADHD Rating Scale—5 for Children and Adolescents, Home Version. It supported the literature on reliability and validity indices of the ADHD Rating Scale-5. We also revealed that the scale can be reliably used in the Sudanese context.

## Data availability statement

The raw data supporting the conclusions of this article will be made available by the authors, without undue reservation.

## Ethics statement

The studies involving humans were approved by the Institutional Ethics Committee in Sudan University of Science and Technology. No. DSR – IEC – 02 -1- 2023. The studies were conducted in accordance with the local legislation and institutional requirements. Written informed consent for participation in this study was provided by the participants’ legal guardians/next of kin.

## Author contributions

MA: Writing – original draft, Writing – review & editing. DB: Writing – original draft, Writing – review & editing. AAl: Writing – original draft, Writing – review & editing. SB: Writing – original draft, Writing – review & editing. RA: Writing – original draft, Writing – review & editing. AAb: Writing – original draft, Writing – review & editing. NA: Writing – original draft, Writing – review & editing. HA: Writing – original draft, Writing – review & editing.

## Footnotes

1 Madani A. Validation of the ADHD scale for basic education students in Khartoum. University of Khartoum, Sudan (2007). Unpublished master’s thesis.

2 Fathi J. Behavioral disorders (aggressive behavior, hyperactivity, rebellion from school) among basic education students: a comparative study of public, private, and model schools in Khartoum. Omdurman Islamic University, Sudan (2010). Unpublished master’s thesis.

3 Khalaf Allah K. Learning difficulties among basic education students in Khartoum and its relation to ADHD. Al-Neelain University, Sudan (2011). Unpublished doctoral dissertation.

4 Mohammed H. Impact of attention deficit hyperactivity disorder on the parents of the affected children in Khartoum. University of Gezira, Sudan (2014). Unpublished master’s thesis.
